# Processing political misinformation: comprehending the Trump phenomenon

**DOI:** 10.1098/rsos.160802

**Published:** 2017-03-01

**Authors:** Briony Swire, Adam J. Berinsky, Stephan Lewandowsky, Ullrich K. H. Ecker

**Affiliations:** 1 School of Political Science, Massachusetts Institute of Technology, 77 Massachusetts Avenue, E53-470, Cambridge, MA 20139USA; 2 School of Psychological Science, University of Western Australia (M304), Perth 6009, Australia; 3 School of Experimental Psychology and Cabot Institute, University of Bristol, 12a Priory Road, Bristol BS8 1TU, UK

**Keywords:** misinformation, continued influence effect, belief updating, motivated cognition, source credibility

## Abstract

This study investigated the cognitive processing of true and false political information. Specifically, it examined the impact of source credibility on the assessment of veracity when information comes from a polarizing source (Experiment 1), and effectiveness of explanations when they come from one's own political party or an opposition party (Experiment 2). These experiments were conducted prior to the 2016 Presidential election. Participants rated their belief in factual and incorrect statements that President Trump made on the campaign trail; facts were subsequently affirmed and misinformation retracted. Participants then re-rated their belief immediately or after a delay. Experiment 1 found that (i) if information was attributed to Trump, Republican supporters of Trump believed it more than if it was presented without attribution, whereas the opposite was true for Democrats and (ii) although Trump supporters reduced their belief in misinformation items following a correction, they did not change their voting preferences. Experiment 2 revealed that the explanation's source had relatively little impact, and belief updating was more influenced by perceived credibility of the individual initially purporting the information. These findings suggest that people use political figures as a heuristic to guide evaluation of what is true or false, yet do not necessarily insist on veracity as a prerequisite for supporting political candidates.

## 1. Introduction

Individuals from opposing sides of the political spectrum often disagree over what is fact and what is fiction. While both conservatives and liberals aim to be well informed, even empirical information that seems straightforward can lead to discord [1]. For example, people perceive unemployment, inflation and crime rates to be lower when their preferred party is in power [2]. Partisanship clearly influences the way people process information, but the exact cognitive mechanisms that underlie these differences are still being debated [3–5]. In this study, we focus on source credibility. Individuals have limited time and cognitive resources to comprehend complex topics such as policy or current affairs, and may therefore use the perceived credibility of political figures as a heuristic to guide their evaluation of what is true or false. For instance, Republicans and Democrats are likely to assess the veracity of a statement differently depending on whether it comes from a favoured politician [6].

To study how individuals evaluate whether political information is true or false, we first examined the impact of source credibility on the initial assessment of information veracity. To this end, we used statements from perhaps the most polarizing political figure of recent times, President Trump. As these experiments were conducted prior to his election and inauguration, we henceforth refer to the him as ‘Donald Trump’, or ‘Trump’. Second, we investigated the impact of source credibility on the corrective effect of retracting misinformation and affirming factual statements.

### 1.1. The continued influence effect

False information continues to influence memory and reasoning even after credible corrections; this has been termed the *continued influence effect* of misinformation [7–9]. Once information is assumed to be true, this conviction is subsequently difficult to change. The continued influence effect occurs even with non-politicized misinformation and at least in part reflects the inherent difficulty of updating one's mental model of an event or a causality [10,11]. However, ongoing reliance on corrected misinformation becomes an even greater problem when the misinformation conforms to a person's pre-existing belief and supports their ideological worldviews, whereas the correction runs counter [12,13]. Once an individual feels personally connected to information, their ideology and values influence how that information is processed [14,15]; this is known as motivated reasoning or motivated cognition.

### 1.2. Motivated cognition

There is an extensive literature on motivated cognition that suggests individuals are more critical when evaluating information that is counter to their beliefs than belief-congruent information, and conclusions that people reach are likely to be consistent with their prior assumptions about how the world functions [16–18]. For example, a classic study by Lord *et al*. [19] found that both supporters and opposers of capital punishment rated studies regarding the death penalty as more convincing when the studies confirmed their existing views. In addition, after receiving mixed evidence comprising both supportive and critical findings, participants' attitudes further diverged—those who initially opposed the death penalty reported opposing it even more, and the reverse occurred for those in support of the death penalty. This illustrates how an individual's worldview can dictate how new information is assessed, legitimizing the preservation of the person's ideological belief system [20].

In the real world, information sometimes turns out to be incorrect and therefore may be subject to revision. Once people have decided that they believe some particular information to be true, they may encounter a correction that challenges their conviction. The extent to which people take heed and change their beliefs based on such corrections may depend on motivated cognition. Specifically, if a correction runs counter to a person's beliefs and worldview, they may be more likely to ignore it, and cling to the original misinformation. For example, when incorrect information arising from a Democratic politician's statement is retracted, Democrats—and particularly those who support the politician—may resist the correction more than their Republican counterparts who have a vested interest in the political figure being incorrect. At worst, a potential outcome of the attempt to correct contentious misinformation is a *worldview backfire effect.* This occurs when an individual feels motivated to defend their belief system, and ironically reports a *stronger* belief in the original misconception after receiving a retraction. For example, worldview backfire effects have been documented with attempts to promote vaccine safety [21], as well as attempts to correct misconceptions regarding anthropogenic climate change or the existence of weapons of mass destruction (WMDs) in Iraq immediately prior to the invasion of 2003 [22,23].

This phenomenon might be especially pronounced among certain individuals. A recent debate in the literature is concerned with the question of whether conservatives are generally more prone to motivated cognition and worldview backfire effects. One school of thought assumes that personality characteristics associated with conservative ideology present a specific susceptibility for motivated cognition. For example, Jost *et al*. [24] suggested that psychological variables such as dogmatism (that is, intolerance of ambiguity, avoidance of complexity and a need for closure) are predictive of conservatism and increase the likelihood that an individual engages in ‘black-or-white’ assessments of information. This tendency to readily decide on information veracity with subsequent resistance to change could lead to greater rejection of factual information for those on the political right relative to moderate and liberal segments of the population [25].

By contrast, Kahan [4] posits that identity-protective motivated cognition occurs equally at both ends of the political spectrum, arguing that conservatives and liberals perform comparably on a measure of information-processing dispositions associated with cognitive biases. Individuals who scored higher on ‘cognitive reflection’—a disposition to engage in effortful processing [26]—were more likely to demonstrate motivated cognition, regardless of partisanship. While the rejection of scientific evidence seems to be primarily associated with conservative ideology [27], the observed asymmetry may not reflect fundamental differences in cognition; rather, it may just be the case that the contested scientific findings happen to challenge primarily the worldview of conservatives rather than liberals [28]. In support of this, Nisbet *et al.* [29] found that liberal participants react in a manner equivalent to conservatives if they encounter liberal-dissonant science messages, for example regarding the efficacy of nuclear power.

In contrast to these backfire effects, Kahan [30] reported no partisan difference for scientific rejection among issues that do not challenge worldviews, such as cell-phone radiation or exposure to high-voltage powerlines. Additionally, Kuklinski *et al*. [31] found that while strong partisans held the least accurate beliefs regarding welfare policy (e.g. the proportion of the federal budget that welfare absorbs), and the highest confidence that these beliefs were accurate, they were not more inclined to reject factual information once corrections were presented. It is therefore possible that party-line differences in the willingness to engage in belief revision are not as pervasive as some research has suggested; there is some evidence that if strong partisans receive quality information, they may be able to interpret it in a similar fashion and update their beliefs to the same extent ([32]; see also [33]).

### 1.3. Source credibility

In addition to motivated reasoning, when people are evaluating whether information is fact or fiction, the source of the information matters a great deal. In general, high-credibility sources are more persuasive and promote greater attitude change than low credibility sources [34]. Additionally, given that attitude homophily—i.e. the extent to which a person perceives similarities between the way they think and another person does—is a key determinant of perceived source credibility, candidate support has substantial impact when estimating the credibility of preferred versus non-preferred political candidates [6]. Two key components of source credibility are (i) expertise—the extent to which the source is *able* to give accurate information—and (ii) trustworthiness—the extent to which the source is *willing* to provide information that the source itself assumes to be correct [35].

When it comes to the efficacy of correcting inaccurate information, it appears that the latter is more important than the former—it is more important that the source of the correction is perceived to be trustworthy than having expertise (U. K. H. Ecker, L. Antonio 2016, unpublished data) [36,37]. This finding suggests that the most effective way to reduce misconceptions is to attribute the correction to a source that the person finds a trustworthy source of information, such as a member of the political party the individual identifies with. On the other hand, there is contrasting evidence suggesting that an *unlikely* source—for example, a Republican correcting another Republican—could be more effective at reducing misconceptions than a source that is expected to provide the corrective information. Thus, a Democrat's belief in misinformation originating from a Republican source may be more strongly reduced by a correction that also comes from a Republican source, rather than a Democrat source [3].

Even if people are able to change their beliefs immediately after a correction, belief change may be fleeting (B. Swire, U. K. H. Ecker, S. Lewandowsky 2016, unpublished data). In this case, worldview and an individual's trust in the veracity of the source may influence the rate of forgetting, and could thus lead to ‘motivated forgetting’ [38]. For example, if misinformation arising from a Democratic politician's statement is retracted, Democrats who support the politician may initially update their belief, but conveniently forget the correction at an accelerated pace over time, thus eventually reverting to their pre-existing beliefs.

Finally, even if it is possible to correct people's misconceptions, it is unclear whether or not such corrections affect candidate support. If an individual acknowledges that a number of a politician's statements are untrue, they should reduce their support to the extent that truthfulness is a desirable trait of a political figure. However, Redlawsk [39] found that participants *increased* their support for candidates whom they endorsed when provided with *negative* information about the candidate. Likewise, Meffert *et al*. [40] found that participants spent more time reading negative stories about candidates they preferred, yet this led to a more *positive* outlook of the candidate. This shows that candidate support ratings are also subject to worldview backfire effects, and it is therefore possible that highlighting misinformation that candidates have disseminated may not result in any loss in support, and could ironically lead to *increased* support.

### 1.4. The case of Donald Trump

It is clear that individuals view the world through a partisan filter; however, the extent to which citizens use partisan cues such as political figures to evaluate the veracity of information and corrections requires further exploration. Donald Trump is an interesting case study for misinformation research, as bipartisan fact-checking media outlets have found that Donald Trump has been particularly prone to inaccuracies [41,42], and for much of the presidential campaign was a divisive figure even among Republicans [43].

While voters are well aware that they encounter politically motivated misinformation during election campaigns, they find it difficult to pinpoint the accuracy of specific messages and are therefore misinformed on a wide array of prominent issues [44]. Donald Trump's popularity, despite the amount of misinformation he distributed, can be explained by either the notion that (i) people believe that his assertions are true (partially because they see Donald Trump as a trustworthy source of information) and they avoid or resist the many corrections available in the public sphere (partially based on motivated cognition), or alternatively (ii) the public is aware that Donald Trump is spreading misinformation, but does not insist on veracity as a prerequisite for their support of a candidate. In this study, we explored these possibilities through several means. First, we tested whether the public believes misinformation spread by a polarizing source, and whether such information can be effectively corrected. We also explored whether a change in belief leads to a shift in voting preferences (i.e. after a credible correction, did people reduce their belief in misinformation yet continued to support Donald Trump?).

Specifically, Experiment 1 investigated (i) whether belief in both misinformation and factual information differs depending on whether or not the information is associated with a polarizing source (i.e. Donald Trump); (ii) whether the impact of corrections/affirmations differs when support for the polarizing source of the original information is taken into account; and (iii) whether belief change is sustained over time. Experiment 2 tested whether the impact of corrective/affirmative explanations is moderated by partisanship (i.e. stating that a correction/affirmation stems from a Democratic, Republican or non-partisan source).

## 2. Experiment 1

Experiment 1 was conducted in November 2015 prior to the Iowa caucus, when 13 other candidates apart from Donald Trump were still viable options (these candidates were Jeb Bush, Ben Carson, Chris Christie, Ted Cruz, Carly Fiorina, Jim Gilmore, Lindsay Graham, Mike Huckabee, John Kasich, George Pataki, Rand Paul, Marco Rubio and Rick Santorum). The experiment featured actual statements made by Donald Trump on the campaign trail in 2015. Some of these statements were inaccurate and others were factual. When these statements were presented to participants, they were either explicitly attributed to Trump or presented without attribution. The objectively false statements were then corrected, and the true statements were affirmed, with a brief explanation. Participants rated their belief in the statements both before and after the corrective/affirmative explanation; the second rating was either immediate or following a one-week delay.

**Figure 1.**
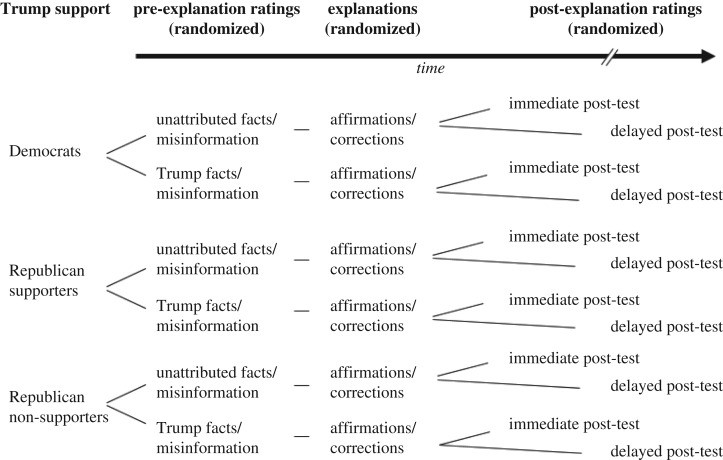
Design schematic of Experiment 1.

To tease apart partisanship from candidate advocacy, we separated Republican participants into those who supported Trump and those who did not. This step is somewhat rare in studies of political cognition, but given the polarizing nature of Trump's candidacy *within* the Republican party at the time of the study, we felt it was inappropriate to mix these two groups. The study thus used a 2 × 2 × 2 × 3 design—type of item (misinformation versus fact) was a within-subjects factor, and the between-subjects factors were the source of information (Trump versus unattributed), study-test retention interval (immediate versus delayed) and Trump support (Democrat versus Republican non-supporters versus Republican supporters). See figure 1 for a schematic diagram of the experimental design. Our prime dependent variable was participants' belief in the inaccurate and factual statements measured on an 11-point scale, as well as participants' self-reported support for Donald Trump.

We hypothesized that participants would use Donald Trump as a cue to evaluate information veracity: we expected that Republican Trump supporters would increase belief in both misinformation and factual statements if they were attributed to Donald Trump, and Democrats and Republican non-supporters would decrease their belief. We also hypothesized that explanations would have a limited effect and would be less sustained over time when they ran counter to participants' expectations arising from their affiliation (i.e. when Republican supporters encountered corrections of Trump's misinformation or Democrats and Republican non-supporters encountered affirmations of Trump's true statements). Lastly, we hypothesized that voting preferences would increase or not change, even if participants reduced belief in misinformation (or increased belief in facts) attributed to Trump.

### 2.1. Method

#### 2.1.1. Participants

Participants were 2023 US residents recruited through Amazon.com's Mechanical Turk. Republican participants who had recently taken part in previous studies from the Massachusetts Institute of Technology's Political Experiments Research Laboratory were invited to participate. We adopted this oversampling strategy due to the relative scarcity of Republicans within the Mechanical Turk population. Participants were paid 85 cents and an additional 50 cents in the one-week delayed condition. They were excluded from the analysis if they did not complete all parts of the study (*n* = 247).^1^ The final sample included *N* = 1776 participants, with 884 males and 892 females in the age range of 19–78 years, with a mean age of *M* = 35.73 (s.d. *=* 11.41).

#### 2.1.2. Stimuli

Four inaccurate statements and four factual statements made by Donald Trump on the campaign trail prior to 1 October 2015 were compiled by the authors. The Trump condition explicitly stated that Donald Trump was the propagator of the information, while the unattributed condition presented the information without specifying its source. Corrections and affirmations of equal length (i.e. two to three sentences) were created; each explanation explicitly referenced a reputable source. Sources were chosen to be non-partisan (e.g. the ‘Danish Epidemiology Science Centre’ or the ‘US Bureau of Labor Statistics’). An example misinformation item with its corresponding correction can be found in table 1 (see appendix A for the complete list of items). Explanations consisted of four segments: (i) the participant was reminded of the initial item; (ii) the veracity was presented; (iii) information was given as to why the statement was true or false and (iv) the participant was given a reminder of their initial belief rating.

**Table 1.** Examples of Trump and unattributed misinformation with corresponding corrections.

	misinformation	correction
Trump	Donald Trump said that vaccines cause autism.	Donald Trump said that vaccines cause autism.
	On a scale between 0 and 10, do you believe Trump's statement to be true?	**This is false.**
		There is strong consensus in the scientific community that vaccines are not linked to autism. For example, one study by the Danish Epidemiology Science Centre tracked all children born in Denmark from 1991 to 1998 and concluded that there was no increase in the rate of autism for vaccinated as opposed to non-vaccinated children.
		You previously rated this statement *x* out of 10 (0 = definitely false, 10 = definitely true)
unattributed	Vaccines cause autism.	Vaccines cause autism.
	On a scale between 0 and 10, do you believe this statement to be true?	**This is false.**
		There is strong consensus in the scientific community that vaccines are not linked to autism. For example, one study by the Danish Epidemiology Science Centre tracked all children born in Denmark from 1991 to 1998 and concluded that there was no increase in the rate of autism for vaccinated as opposed to non-vaccinated children.
		You previously rated this statement *x* out of 10 (0 = definitely false, 10 = definitely true).

#### 2.1.3. Procedure

After reviewing a University of Western Australia and Massachusetts Institute of Technology approved consent form, participants took the survey through Qualtrics.com. They were first presented with general demographic and political-ideology questions. Participants who did not identify with a party, but indicated that they leaned towards a particular party were classified as partisans [45]. This was followed by questions regarding the likelihood of voting for candidates in the 2016 Presidential campaign. The candidates were Donald Trump, Ben Carson, Hillary Clinton and Bernie Sanders, who were the front-runners at the time the survey was conducted. Participants' feelings towards the candidates were also measured using the ‘candidate-feelings thermometers’ employed in the American National Elections Study. These entail asking participants to rate how favourably and warm they feel towards the person; ratings between 0 and 50 degrees are taken to indicate they do not feel particularly warm, and ratings between 50 and 100 are taken to indicate they do feel favourably and warm towards the candidate.

Participants were presented with the eight statements in a randomized order; participants received either all statements attributed to Donald Trump or alternatively presented without source specification. After rating the extent to which they believed each item on a 0–10 scale, participants received an explanation for each item as to whether it was true or false.^2^ Participants then moved directly to the test phase if they were in the immediate post-test group. This involved re-rating belief in all eight statements in random order, as well as re-rating candidate support and feelings towards the candidates. In the delayed post-test condition, participants were instead re-contacted after one week and given the opportunity to complete the test phase.

### 2.2. Results

Of the 1776 participants, 1015 identified as Democrats and 535 identified as Republicans. The 226 participants who had no political affiliation were omitted from the following analyses. Of the Republicans, 323 were classified as Trump supporters as they scored 5 or more (out of 10) on the likelihood to vote for Trump measure, and the 212 participants who scored less than 5 were classified Trump non-supporters. There were 99 Democrats who supported Trump—all main effects and interactions of the following analyses were replicated if these participants were omitted from the analyses.

First, Trump support groups were compared on demographic measures. A one-way ANOVA indicated that age was different between groups, *F*_2,1547_ = 26.03; *p* < 0.001; MSE = 128; $\eta_{p}^{2}=0.03$. Democrats are younger than both Republican groups, *F*_1,1547_ = 46.82; *p* < 0.001; MSE = 128. Next, a one-way ANOVA indicated that education was different between groups, *F*_2,1547_ = 12.29; *p* < 0.001; MSE = 1.48; $\eta_{p}^{2}=0.01$. Planned comparisons revealed that Republican non-supporters were significantly more educated than Democrats, *F*_1,1547_ = 4.51; *p* = 0.034; MSE = 1.48, yet Democrats were significantly more educated than Trump supporters, *F*_1,1547_ = 8.82; *p* = 0.003; MSE = 1.48. Finally, a Pearson *χ*^2^ test revealed there were no gender differences between groups, *χ*^2^ = (3, *N* = 1776) = 2.24, *p* = 0.489. The following analyses remained statistically significant when controlling for education and age using factorial ANCOVAs (unless indicated otherwise).

#### 2.2.1. Pre-explanation belief scores

**Figure 2.**
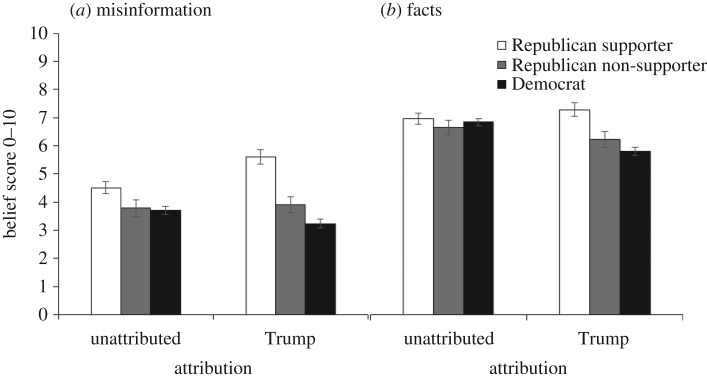
(*a*,*b*) Pre-explanation Democratic and Republican belief in statements associated with Trump or presented unattributed. Error bars denote 95% confidence intervals.

Pre-explanation belief scores partitioned by Trump support are shown in figure 2. Figure 2*a* shows the misinformation, and *b* shows the facts. We further split the sample into those respondents who received statements without source attribution and those who received statements attributed to Trump. For both misinformation and factual statements, Trump attribution was associated with lower belief in the statements among Democrats and greater belief among Republican supporters of Trump. Among Republican non-supporters, a Trump attribution did not affect belief in the misinformation, but did reduce belief in factual statements.

A 2 × 3 factorial ANOVA was performed on the misinformation pre-explanation belief scores. The analysis revealed two significant main effects. The main effect of type of source (unattributed versus Trump), *F*_1,1544_ = 6.12; *p* = 0.013; MSE = 2.60; $\eta_{p}^{2}=0.004$, indicated that Trump attribution influenced belief. The main effect of Trump support (Democrats versus Republican non-supporters versus Republican supporters), *F*_2,1544_ = 116.94; *p* < 0.001; MSE = 2.60; $\eta_{p}^{2}=0.13$, indicated that beliefs of the three groups differed. These main effects were qualified by an interaction between source and Trump support, *F*_2,1544_ = 28.84; *p* < 0.001; MSE = 2.60; $\eta_{p}^{2}=0.04$, reflecting that Trump attribution led to decreased belief for Democrats but increased belief for Trump supporters. Additionally, a planned comparison confirmed that, for Republican non-supporters, misinformation belief was not affected by Trump attribution, *p* = 0.575.

Next, we performed a 2 × 3 factorial ANOVA on the pre-explanation belief scores for the factual statements. The analysis revealed main effects of both type of source, *F*_1,1544_ = 15.96; *p* < 0.001; MSE = 2.25; $\eta_{p}^{2}=0.01$, and Trump support, *F*_2,1544_ = 34.50; *p* < 0.001; MSE = 2.25; $\eta_{p}^{2}=0.04$, as well as an interaction of source and Trump support, *F*_2,1544_ = 25.50; *p* < 0.001; MSE = 2.25; $\eta_{p}^{2}=0.03$. An interaction contrast confirmed that for factual statements, Republican non-supporters believed in the facts less when the information was associated with Trump rather than unattributed, whereas the Republican supporters expressed greater belief in statements made by Trump, *F*_1,1544_ = 8.03; *p* = 0.005; MSE = 2.25. A planned comparison revealed that Democrats believed the statements less if attributed to Trump, *F*_1,1544_ = 119.61; *p* < 0.001; MSE = 2.25. Thus, Trump support influenced the perceived truth of the information.

#### 2.2.2. Post-explanation belief scores

**Figure 3.**
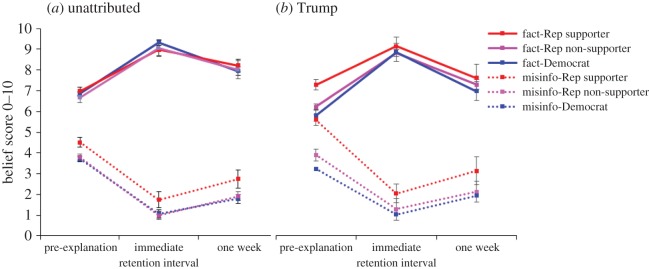
(*a*,*b*) Belief in Trump and unattributed misinformation and facts over time, across Trump support groups and source conditions. Rep, Republican; misinfo, misinformation. Dotted lines show misinformation items. Error bars denote 95% confidence intervals.

The general trend and the full trajectory of belief change over time are shown in figure 3. Figure 3*a* shows the unattributed condition, and *b* shows the Trump-attributed condition. Immediately after the corrections/affirmations, both Democrats and Republicans showed a substantial amount of belief change, which generally diminished over the course of one week for both misinformation and facts. We found no evidence for backfire effects, as post-explanation belief scores in misinformation remained below pre-explanation levels.

**Figure 4.**
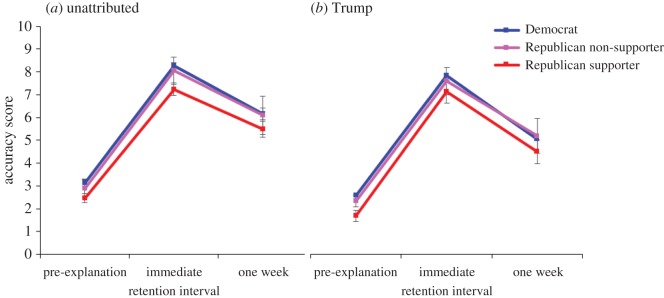
(*a*,*b*) Accuracy scores—misinformation scores subtracted from fact scores—across Trump support and source. Error bars denote 95% confidence intervals.

To simplify the data, we computed total accuracy scores by subtracting participants' misinformation scores from their fact scores. On this measure, the higher the score, the more likely participants were to accurately assume misinformation to be false and factual information to be true. These accuracy scores across conditions are shown in figure 4. A 2 × 2 × 3 factorial ANOVA involving the source, retention interval and Trump support factors was performed on the post-correction accuracy scores. The analysis revealed three significant main effects. The main effect of source, *F*_1,1538_ = 15.42; *p* < 0.001; MSE = 6.93; $\eta_{p}^{2}=0.01$, indicated that Trump attribution was associated with less accurate post-correction beliefs. The main effect of retention interval, *F*_1,1538_ = 183.44; *p* < 0.001; MSE = 6.93; $\eta_{p}^{2}=0.11$, indicated that belief accuracy dropped over the course of a week, and the main effect of Trump support, *F*_2,1538_ = 9.34; *p* < 0.001; MSE = 6.93; $\eta_{p}^{2}=0.01$, indicated that belief accuracy differed by Trump support, with Republican Trump supporters showing the lowest scores overall.

These main effects were qualified by a significant interaction of source and retention interval, *F*_1,1538_ = 3.94; *p* = 0.047; MSE = 6.93; $\eta_{p}^{2}=0.003$, indicating that the influence of Trump attribution changed over time.^3^ From figure 4, we can see that in the immediate condition, Trump attribution does not have a strong influence; over the course of a week, however, participants from all groups seemed to forget the corrective/affirmative explanations at an accelerated rate when the original information was associated with Donald Trump. This was confirmed with a significant planned comparison that focused on the one-week delayed condition that contrasted the Trump-attributed against unattributed condition, and was collapsed over Trump support, *F*_1,1538_ = 15.13; *p* < 0.001; MSE = 6.93. In other words, if the original information came from Donald Trump, after a one-week delay participants had less accurate beliefs, regardless of their affiliation or initial support for Trump.

If the post-explanation misinformation and items are analysed separately, we see similar trends (the full analyses can be found in appendix B). The most prominent differences to the above accuracy score analyses are that (i) misinformation items do not show an interaction of source and retention interval, indicating that unlike the fact scores (where Trump attribution led to less accurate beliefs particularly over time), Trump attribution led to a less accurate belief over both time periods and (ii) fact items additionally show an interaction of Trump support and retention interval, *F*_2,1538_ = 3.44; *p* *=* 0.032; MSE = 2.38; $\eta_{p}^{2}=0.004$. While Democrats over both time periods are worse at updating their belief in the facts if information is attributed to Trump, Republicans immediately update their belief equally in the Trump and unattributed conditions, yet after one week their belief in the Trump-attributed information is less than their belief in the unattributed condition, *F*_1,1538_ = 5.08; *p* *=* 0.0243; MSE = 2.38.

**Figure 5.**
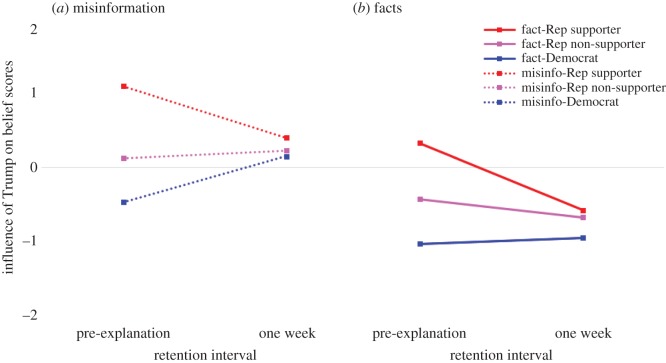
(*a*,*b*) Influence of Trump—Trump-attributed scores subtracted from unattributed scores— across Trump support. Misinfo, misinformation; Rep, Republican. Dotted lines show misinformation items.

To illustrate why accuracy is reduced after one week due to Trump attribution, figure 5 shows the Trump condition subtracted from the unattributed condition—observations above zero indicate that the attribution of a statement to Trump encourages participants to believe the information; values below zero indicate that the attribution of statements to Trump made participants *less* likely to believe in the information. Figure 5*a* shows the misinformation, and *b* shows the facts. The distance from zero indicates the impact that the Trump attribution is having upon the belief scores. Figure 5 highlights the fact that initially, before they receive the correction, participants use their support for Donald Trump as a heuristic for whether information is true or false (i.e. independent of actual veracity, Republican supporters believe Trump information more, Democrats believe Trump information less, and Republican non-supporters are not affected much). However, after one week—regardless of partisanship and level of Trump support—people tend to assume Trump's facts are incorrect, and Trump's misinformation is accurate.

#### 2.2.3. Likelihood-to-vote and feelings-thermometer ratings

**Figure 6.**
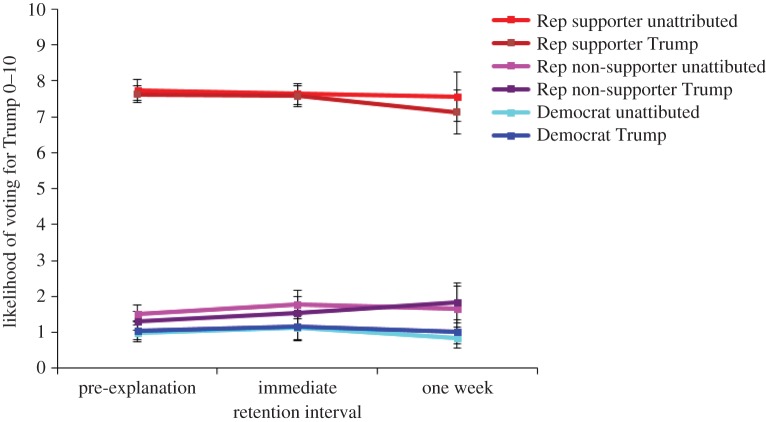
Likelihood-to-vote ratings over time between Trump support and source. Error bars denote 95% confidence intervals. Rep, Republican; Misinfo, misinformation.

Attributing the information to Trump did not influence participants' intention to vote nor their feelings towards the political figure. Figure 6 shows the full trajectory of participants' likelihood to vote for Donald Trump, both prior to and after the corrective/affirmative explanations. To simplify the analysis, the post-explanation scores were subtracted from the pre-explanation scores to create change indices for both the likelihood-to-vote and feelings-thermometer ratings.

A 2 × 2 × 3 factorial ANOVA on the likelihood-to-vote change index revealed two main effects. The main effect of Trump support, *F*_2,1537_ = 13.39; *p* < 0.001; MSE = 1.35; $\eta_{p}^{2}=0.02$, indicated that Republicans were more likely to change their voting preferences than were Democrats. For example, Republican non-supporters increased their support for Trump by 0.22 on the 10-point scale in the immediate condition and by 0.35 after one week, yet Democrats only increased their support by 0.07 in the immediate condition, and decreased their support by 0.01 after one week. The main effect of retention interval, *F*_1,1538_ = 8.00; *p* *=* 0.005; MSE = 1.35; $\eta_{p}^{2}=0.005$, indicated that change was greater after one week than immediately after the explanations.

These main effects were qualified by an interaction of retention interval and Trump support, *F*_2,1537_ = 9.06 *p* < 0.001; MSE = 1.35; $\eta_{p}^{2}=0.01$, indicating that change in voting preferences differed between Trump support groups over time. Republican supporters slightly reduced their likelihood of voting for Trump and Republican non-supporters slightly increased their likelihood. As there was no main effect or interaction of source, it can be assumed that these differences can be attributed to natural fluctuation of voting preferences over time rather than the explicit association of information to Donald Trump. The likelihood-to-vote trend was mimicked by the feelings-thermometer ratings (see appendix C for the figure and analysis).

Finally, 48 pairwise correlations were calculated for Democrats, Republican supporters and Republican non-supporters to investigate whether belief change in misinformation or factual statements was associated with (i) a change in likelihood to vote or (ii) feelings towards Trump over time for each retention interval and source. Using a Bonferroni-adjusted alpha level of 0.001, two correlations were significant, revealing that the more Democrats reduced their belief in Trump-attributed misinformation, the more they reduced their feelings and likelihood of voting for Trump one week post-explanation (*r* = 0.36 and *r* = 0.33, respectively). This could reflect the fact that Democrats who reduce misconceptions attributed to Trump view him less favourably after one week, or alternatively, that those who do not like Trump report that they believe him less after one week. The absence of significant correlations for the remaining Democratic and Republican groups indicated that their intentions to vote and feelings towards Trump were independent of belief change.

### 2.3. Discussion

Experiment 1 revealed several notable findings. First, when initially evaluating the veracity of both misinformation and factual statements, Republican supporters of Trump believed the information more when it was attributed to Trump, whereas the opposite occurred for Democrats. Republicans who did not support Trump also believed less in facts associated with Trump (but not to the same extent as Democrats), while their belief in the misinformation was not affected by information source. Overall, the Trump attribution did indeed colour people's assessment of information veracity, dictating how valid they perceived it to be.

Second, there was a large bipartisan shift in belief post-explanation, indicating that all members of the political spectrum are capable of substantial belief change when sound non-partisan explanations are presented. However, after a one-week delay, participants partially ‘re-believed’ in the misinformation and partially forgot that factual information was true. Thus, even if individuals update their beliefs temporarily, explanations regarding both fact and fiction seemingly have an expiration date (cf. B. Swire, U. K. H. Ecker, S. Lewandowsky 2016, unpublished data). People revert to their original assumptions, highlighting that once inaccurate information is in the public sphere, it is difficult to permanently correct, and reservations regarding factual information are likewise challenging to permanently overcome.

From the pre-explanation belief scores, we know that Republican Trump supporters were predisposed to assume that information attributed to Trump was correct, regardless of its actual veracity. One week after the explanations, this bias continued for the misinformation items, but for factual items participants became *less* likely to think that Trump's statements were true. Similarly, Democrats—who are predisposed to assume that information attributed to Trump is incorrect—continued to exhibit bias for factual items, but were more likely to think Trump's misinformation was true. It thus seems as if participants on both sides of the spectrum took into account their Trump-related biases but overcorrected for them: Republican supporters overcorrected by assuming that Trump's facts were false, and Democrats overcorrected by assuming that Trump's misinformation was true.

Third, Republican Trump supporters showed the highest level of post-explanation belief in misinformation in both Trump and unattributed conditions. This may reflect that only so much belief revision is possible (as their pre-explanation misinformation belief was also at a higher level), or alternatively that Republican Trump supporters were less inclined to believe our corrections.

Fourth, it was noteworthy that if the original information came from Donald Trump, after an explanation participants were less able to accurately label what was fact or fiction in comparison to the unattributed condition, regardless of their support for Trump. This was particularly the case for fact items after a delay, where even the Republican groups were less likely to think that the true information was indeed accurate if attributed to Trump.

Finally, while Republican supporters did update their beliefs when presented with corrections of misinformation, they did not change their voting intentions nor feelings towards Trump when the misinformation was attributed to the political figure. The degree that Republican supporters updated their belief that Trump's misinformation was false was not significantly correlated with a change in voting intentions nor feelings towards Trump. This suggests that the public, or at least Trump supporters, are not overly concerned with a candidate disseminating misinformation and seem to be looking to qualities other than veracity.

To test how processing of corrective/affirmative explanations is moderated by explanation source, we ran Experiment 2.

## 3. Experiment 2

**Figure 7.**
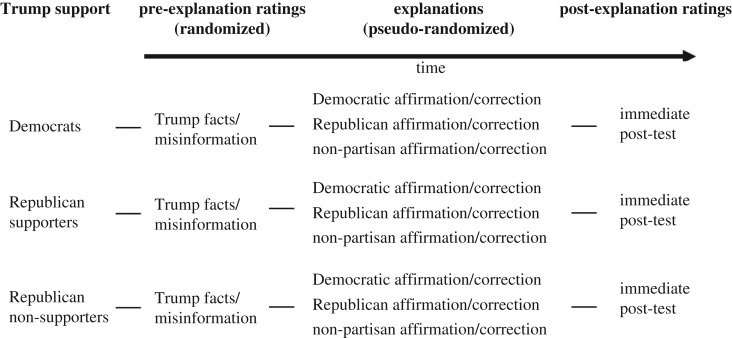
Design schematic of Experiment 2.

Experiment 2 was conducted in July 2016. As in Experiment 1, participants were presented with inaccurate statements and factual statements that Donald Trump mentioned on the campaign trail in 2015, and the objectively false statements were corrected and the true statements affirmed. However, unlike Experiment 1, all statements were attributed to Trump. The other predominant difference between the two experiments was that we varied the nature of the explanations regarding the veracity of the information. In Experiment 2, the same explanations came from different partisan sources. Specifically, we randomized the attribution of the explanation to follow one of three forms: (i) ‘According to Democrats’, (ii) ‘According to Republicans’ or (iii) ‘According to a non-partisan fact-checking website’. Participants rated their belief in the statements both before and immediately after the explanation (though not one week later). The study thus used a 2 × 3 × 3 design, with the within-subjects factors type of item (misinformation versus fact) and explanation source (Democrat versus Republican versus non-partisan), and a between-subjects factor of Trump support (Democrat versus Republican non-supporters versus Republican supporters). See figure 7 for a schematic of this design. Our prime dependent variables were participants' belief in the statements, as well as participants' self-reported support for Donald Trump.

Two potential outcomes were that (i) partisanship-congruent explanations would be more effective than partisanship-incongruent explanations due to greater support and trust in the source (e.g. Democrats being more influenced by a Democratic explanation) (U. K. H. Ecker, L. Antonio 2016, unpublished data) [36,37] or (ii) a Democratic source would be more effective for all participants at affirming Trump's factual statements, and a Republican correction would be more effective at retracting Trump's misinformation, due to the surprise of an unlikely source presenting the explanation [3].

### 3.1. Method

#### 3.1.1. Participants

Participants were 1019 US residents recruited through Survey Sampling International of Shelton, Connecticut. An over-18 population was recruited, targeting the census population for education, gender, age, geography and income, resulting in a diverse national sample. Participants were excluded from the analysis if they did not complete all parts of the study (*n* = 59).^4^ The final sample included *N* = 960 participants, with 456 males and 504 females. The age range was 19–86 years with a mean age of *M* = 41.89 (s.d. *=* 17.96).

#### 3.1.2. Stimuli

As stimuli, we used six of the eight statements from Experiment 1: three inaccurate statements and three factual statements. The corrective/affirmative explanations were pseudo-randomly determined. Specifically, each item was attributed to one of the three different explanation sources (Republican, Democrat and non-partisan) in a counterbalanced manner, but we ensured that participants received all explanation sources during the experiment. This resulted in each participant seeing each of the respective explanation sources for one misinformation and one factual statement.

#### 3.1.3. Procedure

Participants first rated their likelihood to vote for Donald Trump, and were then presented with all six statements in a randomized order. Participants rated the extent to which they believed each item to be true on a 0–10 scale, prior to receiving an explanation for each item as to whether it was true or false (with explanations coming from the three different sources). The test phase involved re-rating belief in all six statements in random order as well as re-rating Trump support immediately after all explanations were presented.

### 3.2. Results

Of the 960 participants, 514 identified as Democrats. Of the 286 Republicans, 186 were Trump supporters and 100 were Trump non-supporters. The 160 participants who had no political affiliation were omitted from the following analyses. There were 81 Democrats who supported Trump—all main effects and interactions of the following analyses were replicated if they were omitted from the analyses. A one-way ANOVA revealed there was a main effect of age, *F*_2,797_ = 4.88; *p* = 0.008; MSE = 328.70; $\eta_{p}^{2}=0.01$, reflecting the fact that Republican non-supporters were younger than Republican supporters and Democrats. The following analyses remained statistically significant when controlling for age using repeated measures general linear models. There were no gender differences between groups (*p* = 0.121), nor education differences (*p* = 0.346).

#### 3.2.1. Pre-explanation belief scores

**Figure 8.**
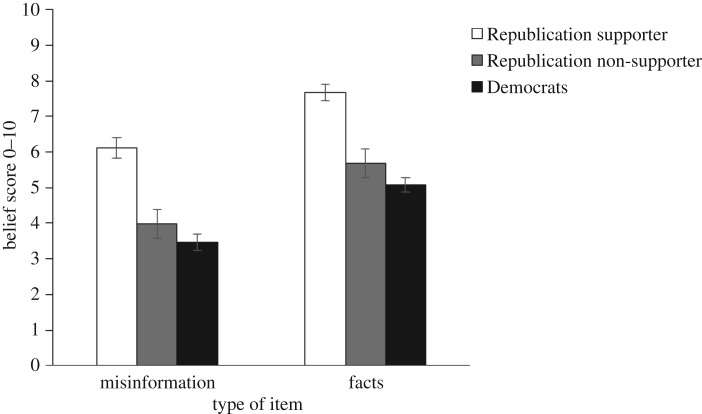
Pre-explanation Democratic and Republican belief in statements associated with Trump. Error bars denote 95% confidence intervals.

Pre-explanation belief scores by Trump support are shown in figure 8. In a clear replication of Experiment 1, the Trump attribution led all participants to support the information to the extent that they supported Trump.

A 2 × 3 repeated measures ANOVA was performed on the pre-explanation belief scores. The analysis revealed two significant main effects. The main effect of type of item (misinformation versus fact), *F*_1,797_ = 322.37 *p* < 0.001; MSE = 2.13; $\eta_{p}^{2}=0.29$, indicated that misinformation was believed less than facts. The main effect of Trump support (Democrats versus Republican non-supporters versus Republican supporters), *F*_2,797_ = 114.49; *p* < 0.001; MSE = 8.27; $\eta_{p}^{2}=0.22$, indicated that pre-explanation belief scores differed by Trump support. Republican supporters clearly believed Trump statements more than the other two groups; a planned comparison also indicated that Republican non-supporters believed the information more than Democrats, *F*_1,797_ = 6.40; *p* = 0.012; MSE = 8.27.

#### 3.2.2. Post-explanation belief scores

**Figure 9.**
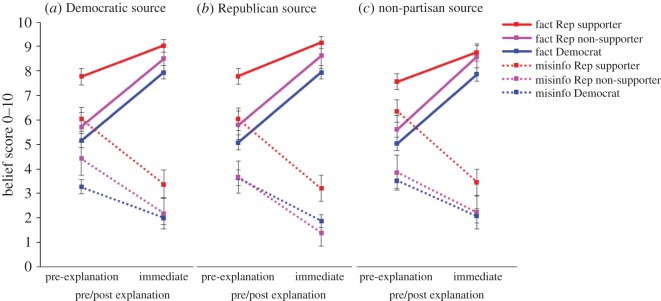
(*a*–*c*) Belief in Trump misinformation and facts after partisan explanations. Rep, Republican; Misinfo, misinformation. Dotted lines show misinformation items. Error bars denote 95% confidence intervals.

The general trend and the full trajectory of pre- and post-explanation belief change over time are shown in figure 9. Immediately after the corrections/affirmations, both Democrats and Republicans showed a substantial amount of belief change—belief in misinformation reduced and belief in factual information increased. Partisanship and Trump support were far better predictors of the extent of belief updating than the explanation source.

A 3 × 3 repeated measures ANOVA involving explanation source (Democrat versus Republican versus non-partisan) and Trump support (Democrat versus Republican supporter versus Republican non-supporters) was performed on the post-explanation misinformation belief scores. The analysis revealed a main effect of Trump support, *F*_2,797_ = 19.15; *p* < 0.001; MSE = 20.72; $\eta_{p}^{2}=0.05$, indicating that groups differed in their belief, with Republican supporters believing in the misinformation more than Republican non-supporters and Democrats. There was also a main effect of explanation source, *F*_2,1594_ = 6.01; *p* = 0.003; MSE = 4.81; $\eta_{p}^{2}=0.007$, showing that a Republican correction reduced belief to a greater extent than the Democratic or non-partisan corrections. However, it must be noted that this is a small effect size and should be interpreted with caution.

To explore the observed trend that post-correction misinformation belief seemed to depend on the correction source in Republican non-supporters more so than in Democrats and Republican supporters, we ran an interaction contrast. Contrasting Republican non-supporters against the pooled Democrats and Republican supporters, and the Republican correction against the pooled Democrat and non-partisan corrections, revealed a significant effect, *F*_1,797_ = 4.79; *p* = 0.029; MSE = 4.68. It appears that misinformation belief was lowest after a Republican correction (versus Democrat/non-partisan correction) in Republican non-supporters, *F*_1,797_ = 9.69; *p* = 0.002, whereas there were no effects of correction source on post-correction misinformation belief in Democrats or Republican supporters (all *F*_1,797_ < 1.27; *p* > 0.257). However, as these were post hoc analyses of a marginal effect, they too should be interpreted cautiously.

A 3 × 3 mixed ANOVA restricted to the post-affirmation fact belief scores revealed a main effect of Trump support, *F*_2,797_ = 19.96; *p* < 0.001; MSE = 12.70; $\eta_{p}^{2}=0.05$, indicating that Republican supporters were more accurate for fact belief than Republican non-supporters and Democrats.

#### 3.2.3. Likelihood to vote

**Figure 10.**
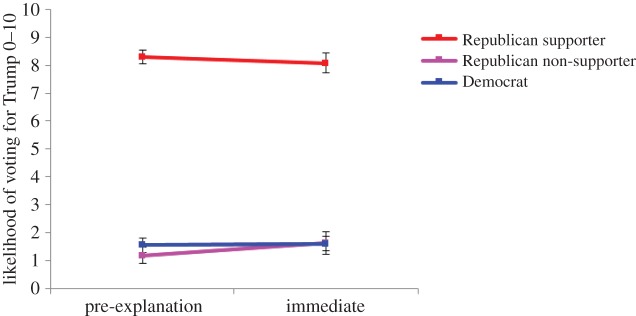
Likelihood of voting for Trump across Trump support groups. Error bars denote 95% confidence intervals.

Figure 10 shows the full trajectory of participants' likelihood to vote for Donald Trump, both prior to and after the corrective/affirmative explanation. Explanations regarding Trump statements did not greatly influence participants' intention to vote. As in Experiment 1, to simplify the analysis, post-explanation scores were subtracted from the pre-explanation scores to create a vote change index.

A one-way ANOVA on the likelihood to vote for Trump change index revealed a main effect of Trump support, *F*_2,797_ = 8.23; *p* < 0.001; MSE = 1.68; $\eta_{p}^{2}=0.02$, indicating that change differed between groups. Republican non-supporters increased their likelihood to vote for Trump significantly more (by +0.44) than Democrats and Republican supporters (who shifted their rating by +0.05 and −0.21, respectively), *F*_1,797_ = 7.72; *p* = 0.006; MSE = 1.67.

Analogous to Experiment 1, pairwise correlations were calculated for all Trump support groups to investigate whether belief change in misinformation or factual statements was associated with a likelihood to vote for Trump. As in Experiment 1, intentions to vote for Trump were largely independent of belief change. However, using a Bonferroni-adjusted alpha level of 0.008, three correlations were significant: Democrats were shown to (i) reduce their likelihood to vote for Trump the more they reduced their belief in Trump-attributed misinformation (*r* = 0.13), as well as (ii) increase their likelihood to vote for Trump the more they increased their belief in Trump-attributed facts (*r* = 0.18). Somewhat ironically, (iii) Republican Trump supporters reduced their likelihood to vote for Trump when they increased their belief in the Trump-attributed fact items (*r* = −0.24).

### 3.3. Discussion

Experiment 2 primarily investigated whether partisanship-congruent explanations were more effective than partisanship-incongruent explanations, or whether an unexpected explanation source would be more effective. Pre-explanation findings of Experiment 1 were replicated, as Republican supporters believed in the Trump-attributed misinformation and factual information to a greater extent than both the Republican non-supporters and Democrats.

Post-explanation, we found that the partisanship congruence of explanations did not have as large an impact as hypothesized, and post-explanation belief was rather dictated by the group membership of the individual (i.e. whether the participant was a Democrat, Republican non-supporter or Republican supporter). However, Republican non-supporters were somewhat more likely to update their misinformed beliefs if a correction was attributed to a Republican source. It is possible that a respected explanation source is particularly helpful when the initial information is from a source that is not respected, although this effect did not extend to true statements.

Finally, the increase in the Republican non-supporters' post-explanation likelihood-to-vote ratings could reflect a backfire effect—it is plausible that Republican non-supporters do not wish to be nudged by explanations that could be perceived as liberal, thus leading them to further support the Republican figure. However, as Experiment 2 did not have an unattributed control condition for comparison (as Experiment 1 did), it is uncertain whether or not this shift was due to the Trump attribution of the corrections.

## 4. General discussion

The present research aimed to determine whether belief in misinformation and factual information depended on whether or not it stemmed from a politically polarizing source, and whether it could be successfully corrected or affirmed. To this end, we presented participants with both inaccurate and factual statements made by Donald Trump on the campaign trail. Experiment 1 allowed us to investigate whether people use their support in political figures as a heuristic to guide their assessment of the veracity of this information, and Experiment 2 addressed whether partisanship-congruent explanations were more effective than partisanship-incongruent explanations. By keeping the content of the initial information and explanations stable across conditions, we were able to provide an accurate measure of a source's impact upon information processing.

### 4.1. Pre-explanation belief scores

We found that participants' opinion of Donald Trump influenced their assessment of information, that is, how valid they perceived it to be. The graded nature of information belief when it was attributed to Trump in comparison to the unattributed condition (i.e. Democrats decreasing, Republican supporters increasing, Republican non-supporters not affected as much) fits well with the graded intention to vote for Donald Trump, as revealed in Experiment 1. These findings are consistent with the findings from the literature regarding source credibility [34]. Given that attitude homophily is a crucial component of source credibility [6], coupled with the notion that higher source credibility results in an increased perception of information credibility [46], it is reasonable that political figures such as Donald Trump act as a heuristic when evaluating the veracity of information.

Democrats showing lower levels of belief when information is attributed to Trump could reflect rational updating that takes the experienced base rates into account. However, this could also be an occasion where Democrats demonstrate equal biases to those of Republicans. While Republicans increased belief in inaccurate information if it came from a source they regard as trustworthy, Democrats indicated lower fact belief if the information came from a source they did not regard as trustworthy. Some of the true items used in this study are more aligned with traditional liberal ideology (e.g. that the USA spent $2 trillion on the war in Iraq), indicating that this effect holds even when processing factual information that could be considered worldview-congruent. This supports Kahan's [4] stance that biases such as motivated cognition could occur at both ends of the political spectrum, while running counter to the notion that people who hold right-wing ideology are more susceptible to motivated cognition in general. Our paper therefore contributes to mounting literature that all individuals—regardless of partisanship—are biased by their own worldview, rather than there being fundamental differences in cognition between people with differing political values [29,30,33,47–49].

### 4.2. Post-explanation Trump attribution

Intriguingly, even when Trump statements were followed by credible explanations that ought to induce sustained knowledge revision and belief updating, in all groups there was a greater level of inaccuracy in comparison to the unattributed condition. This was particularly the case with regard to factual statements over the long term.

Republicans and Democrats seemed to take into account their Trump-related biases and overcorrected for them one week after the explanations: Republican supporters by assuming that Trump's facts were false and Democrats by assuming that Trump's misinformation was true. There is precedent for such meta-cognitive effects in the political information-processing literature. *Overcorrection* has been seen to occur for mood-related biases when people assume their feelings are affecting their judgement and attempt to correct for their influence [50]. For example, Isbell & Wyer [51] found that participants rated political figures less favourably when participants were happy than when they were not, in an attempt to adjust for what they perceived to be an irrelevant affective influence. This overcorrection for biases appears to also influence the judgement of veracity when it comes to correcting misinformation and affirming factual information that stems from a polarizing source.

It is important to highlight that Trump attribution has a relatively small effect size in comparison with the common effects of the retention interval in the post-explanation analyses. The consistency in belief updating and forgetting over time perhaps reflects that partisan effects are not as consequential as more general cognitive consequences such as the reversion to original assumptions over time.

### 4.3. Explanation source

Different explanation sources did not have as large an impact as hypothesized. It is noteworthy in itself that the explanation source did not have as large an impact as the support of the person purporting the initial information. While Berinsky [3] found that corrections from an unlikely source aided belief updating, this was when the to-be-corrected information was specifically counter to the traditional stances of a political party, for example, when Republicans debunked rumours regarding health care. It is possible that our amalgamation of items was not sufficiently in opposition to the core values of the Republican party to replicate these results. While it seemed that Republican non-supporters reduced their misinformation belief most following a Republican correction, it is necessary to replicate these results due to the post hoc nature of the analysis.

### 4.4. Worldview backfire effects

There was no evidence for a worldview backfire effect in either experiment, as post-explanation misinformation belief scores remained below pre-explanation levels. In 2005, Nyhan & Reifler [23] found a backfire effect in conservatives when trying to correct the belief that weapons of mass destruction were found in Iraq. Yet in 2006, this effect was not replicated—the correction led conservatives to appropriately update their belief. The authors argued that, between 2005 and 2006, conservatives came to place less importance on the war, suggesting that backfire effects may only occur when an issue is strongly and currently connected with an individual's political identity. In the present case, perhaps not all four pieces of misinformation resonated strongly enough with Republicans to create a notable backfire effect. The present pattern—obtained using a variety of real-world items rather than relying on only one contentious topic (as previous studies have [21–23])—suggests that worldview backfire effects are not the norm and may only occur under very specific circumstances.

### 4.5. Voting preferences

While it is possible that the observed changes in voting preferences between pre- and post-explanation are due to the presentations of the corrections and affirmations, it appears that the negative political ramifications of disseminating misinformation are limited. Belief change in Trump-attributed misinformation remained uncorrelated with a change in voting intentions and feelings towards Trump. Many individuals, and indeed political scientists, did not predict the success of Donald Trump [52,53]. This study contributes one further piece of the puzzle as to why his success was sustained: spreading misinformation did not hinder his candidacy, and even if misinformation was exposed, this did not reduce voting preferences or positive feelings. This could reflect that, to a certain extent, people expect politicians to make inaccurate statements [54], thus they are not overly concerned when this expectation is met. Moreover, in the context of this study, providing an equal number of misinformation and factual items could have both reduced and boosted candidate support. Although people's opinions of a political candidate should ideally not increase if they hear the candidate made a factual statement—this should be an expectation rather than an added benefit—the equal presentation of misinformation and facts could explain the null effect. An avenue for future research would be to vary the proportion of true and false statements from the political figure that are provided to participants.

Understanding Donald Trump's popularity, despite the degree of misinformation he has distributed [41,42], is an interesting case study of American politics. However, it is uncertain to what extent the findings of the current experiments are in fact a ‘Trump phenomenon’. While he is perhaps a good candidate for the study of misinformation, political misinformation is common in the political arena [1]. To test whether the present findings are generalizable beyond Donald Trump, this experiment should be replicated with a Democratic and a different Republican political figure. Another potential barrier to generalizability is that the participants from Experiment 1 were Mechanical Turk workers. However, several studies have found that this population yields high-quality data, comparable to other convenience samples such as university students [55,56], and Experiment 2 replicated Experiment 1's data trends in a more diverse sample.

There are many possible explanations for why Americans voted for Donald Trump in the primary and the general election: factors such as his perceived business acumen, his economic or immigration policies, or perhaps the fact that he was not a career politician increased his appeal [57,58]. We cannot speak to these possibilities. This study illustrates that something other than veracity accounted for his success, as supporters did not change their voting intentions even if they altered their beliefs about the truth of his statements. If spreading falsehoods does not discredit character, it is perhaps not surprising that many individuals rallied behind him on election day [59,60]. According to Ramsay *et al*. [44], 91% of voters said that information in campaigns sometimes seemed misleading or false, yet struggled to pinpoint exactly what is fact and what is fiction. The real-world consequences of this study suggest that politicians can seemingly spread misinformation without dramatic negative consequences of losing supporters—the results of the 2016 Presidential election are consistent with this interpretation. It thus appears that it is possible to appeal through the art of rhetoric and demagoguery rather than necessitating cohesive arguments constructed of logic and fact.

## Figures and Tables

**Figure 11.**
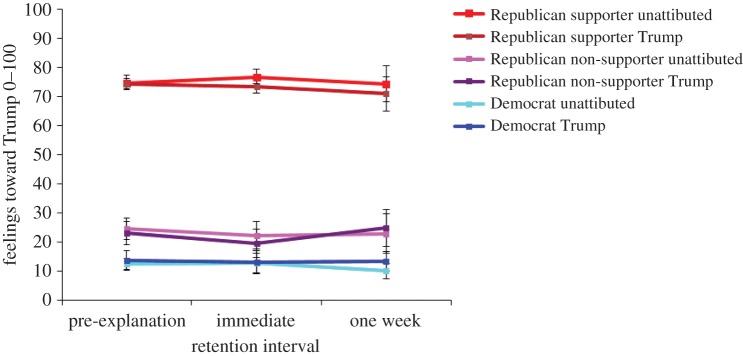
Feelings-thermometer scores over time across partisanship and sources. Error bars denote 95% confidence intervals.

## Footnotes

1 Of the excluded participants, 94% were in the one-week retention interval. A Pearson *χ*^2^-test indicated that neither Trump support, *χ*^2^ = (3, *N* = 2023) = 1.92, *p* = 0.589, nor source, *χ*^2^ = (1, *N* = 2023) = 0.28, *p* = 0.592, differed between participants who were included and those who were excluded.

2 As all items were presented within-subjects, it could be a concern that participants receiving multiple pieces of corrective information are more vulnerable to social desirability biases. However, a one-way ANOVA on the pre-explanation belief ratings confirmed that their presentation order did not have a significant influence on belief, *F*_7,12425_ = 1.38; *p* = 0.210. Post-explanation belief was likewise not affected by presentation order, *F*_7,12425_ = 1.61; *p* = 0.127.

3 This interaction was only marginally significant when controlling for age, *p* = 0.060.

4 A Pearson *χ*^2^-test indicated that there was no difference in Trump support between participants who were included and those who were excluded, *X*^2^ = (3, *N* = 983) = 5.42, *p* = 0.144.

## Data accessibility

The data are publicly available from the Dryad Digital Repository: http://dx.doi.org/10.5061/dryad.nc571 [61].

## Appendix A

See table 2.

**Table 2.** Trump and unattributed items and their corresponding explanations.

item number	trump item	unattributed item	explanation
misinformation 1	Donald Trump said that there are 30–34 million illegal immigrants residing in the USA	There are 30–34 million illegal immigrants residing in the USA	According to the Department of Homeland security's most recent estimate, the number of illegal immigrants currently residing in the USA is 11.4 million people. In 2014, the Pew Research Center placed this number at 11.3 million people, while the Center for Migration Studies estimated approximately 11.0 million individuals
misinformation 2	Donald Trump said that the real unemployment rate is between 24 and 48%	The real unemployment rate is between 24 and 48%	The US Bureau of Labor Statistics states that the official unemployment rate is 5.5%. There are more lenient measures of unemployment that includes people who have part-time jobs but would prefer full-time work, and people who are not looking for work. If these individuals are included, the unemployment rate only rises to 10.8%
misinformation 3	Donald Trump said that the gross domestic product (GDP) growth rate has never been below zero	The gross domestic product (GDP) growth rate has never been below zero	The GDP growth rate indicates how much a country's production has increased in comparison to the previous year, and is an indicator of a country's economic strength. The US Department of Commerce Bureau of Economic Analysis reports that there were 42 occurrences since 1946 where the US growth rate was below zero
misinformation 4	Donald Trump said that vaccines cause autism	Vaccines cause autism	There is strong consensus in the scientific community that vaccines are not linked to autism. For example, one study by the Danish Epidemiology Science Center tracked all children born in Denmark from 1991 to 1998 and concluded that there was no increase in the rate of autism for vaccinated as opposed to non-vaccinated children
fact 1	Donald Trump said that the US debt is $18 trillion	The US debt is $18 trillion	Each day the US Department of the Treasury releases the exact amount of US debt. The debt currently sits at $18.15 trillion. The public holds $13 trillion, which is debt held by individuals and corporations. A further $5 trillion is intra-governmental debt, which is the government borrowing from federal trust funds
fact 2	Donald Trump said that the US spent $2 trillion on the war in Iraq	The US spent $2 trillion on the war in Iraq	A report by the Watson Institute found that as of 2013, the US spent $1.7 trillion on the war in Iraq. While the appropriations for the war were under $800 billion, the Watson report also included the cost of disability, Defense Department base spending costs and homeland security expenditures attributed to Iraq
fact 3	Donald Trump said that the USA is ranked 26th in the world in education	The US is ranked 26th in the world in education	The Program for International Student Assessment is a test for children 15 years of age. It is administered every three years, and largely focuses on math, reading and science. The most recent test was administered in 2012, when the USA ranked between 24th and 35th on the three measures, scoring below average in each category
fact 4	Donald Trump said that Nabisco, the company that manufactures Oreo cookies, is moving jobs to Mexico	Nabisco, the company that manufactures Oreo cookies, is moving jobs to Mexico	Nabisco is a food snack company that is known for products such as Oreos and Ritz crackers. It announced that it will open a new factory in Mexico, rather than investing the $130 million in their current factory in Chicago. Over the next year, half the workers at the Chicago-based bakery will lose their job, which totals 600 employees

## Appendix B

If the post-explanation items are analysed separately, we see similar trends as to the accuracy score analyses. For the fact items we likewise see the main effects of source, *F*_1,1538_ = 19.79; *p* < 0.001; MSE = 2.38; $\eta_{p}^{2}=0.01$, retention interval, *F*_1,1538_ = 190.48; *p* < 0.001; MSE = 2.38; $\eta_{p}^{2}=0.11$, and an interaction of source and retention interval, *F*_1,1538_ = 9.00; *p* *=* 0.003; MSE = 2.38; $\eta_{p}^{2}=0.006$. In addition, there is a Trump support and source interaction, *F*_2,1538_ = 3.28; *p* *=* 0.038; MSE = 2.38; $\eta_{p}^{2}=0.004$, indicating that Trump support influences the degree to which the Trump attribution influences belief. A planned comparison indicates that Democrats do not update their belief in the factual items to the same extent as the Republican groups if the information is attributed to Trump, *F*_1,1538_ = 5.12; *p* *=* 0.024; MSE = 2.37. There is also an interaction of Trump support and retention interval, *F*_2,1538_ = 3.44; *p* *=* 0.032; MSE = 2.38; $\eta_{p}^{2}=0.004$. While Democrats over both time periods are worse at updating their belief in the facts if information is attributed to Trump, Republicans immediately update their belief equally in the Trump and unattributed conditions, yet after one week belief in the Trump information reduces below that in the unattributed condition, *F*_1,1538_ = 5.08; *p* *=* 0.024; MSE = 2.38.

The post-explanation misinformation items reveal three main effects. A marginal main effect of source, *F*_1,1538_ = 3.78; *p* *=* 0.052; MSE = 3.20; $\eta_{p}^{2}=0.002$, indicating that the Trump attribution led to less accurate belief, and a main effect of Trump support, *F*_2,1538_ = 33.35; *p* < 0.001; MSE = 3.20; $\eta_{p}^{2}=0.04$, indicating that Republican supporters had higher belief in the misinformation than Democrats and Republican supporters, *F*_1,1538_ = 53.00; *p* < 0.001. Finally, a main effect of retention interval, *F*_2,1538_ = 64.50; *p* < 0.001; MSE = 3.20; $\eta_{p}^{2}=0.04$ indicating belief increased over time, all groups forgetting that the misinformation was in fact false. There was no interaction of source and retention interval, indicating that unlike the fact scores (where Trump attribution led to less accurate beliefs particularly over time), the information associated with Trump is considered to be less accurate over both time periods.

## Appendix C

The feelings thermometer scores can be seen in figure 11. A 2 × 2 × 3 factorial ANOVA on the feelings-change index revealed an interaction of retention interval and Trump support, *F*_2,11530_ = 21.67; *p* < 0.001; MSE = 139.37; $\eta_{p}^{2}=0.03$, indicating that Republican non-supporters and Republican supporters changed their feelings towards Trump more than Democrats. Mimicking voting preferences, over the course of a week Republican supporters indicated feeling ‘cooler’ towards Trump, and Republican non-supporters indicated feeling ‘warmer’.
